# Health behaviours, body weight and self-esteem among grade five students in Canada

**DOI:** 10.1186/s40064-016-2744-x

**Published:** 2016-07-16

**Authors:** Xiuyun Wu, Sara F. L. Kirk, Arto Ohinmaa, Paul Veugelers

**Affiliations:** School of Health and Human Performance, Dalhousie University, Halifax, NS Canada; IWK Health Centre, Halifax, NS Canada; School of Public Health, University of Alberta, 350 University Terrace, 8303 - 112 Street, Edmonton, AB T6G 2T4 Canada

**Keywords:** Children, Diet quality, Physical activity, Sedentary behaviour, Self-esteem, Mental health

## Abstract

**Purpose:**

This study sought to identify the principal components of self-esteem and the health behavioural determinants of these components among grade five students.

**Methods:**

We analysed data from a population-based survey among 4918 grade five students, who are primarily 10 and 11 years of age, and their parents in the Canadian province of Nova Scotia. The survey comprised the Harvard Youth and Adolescent Questionnaire, parental reporting of students’ physical activity (PA) and time spent watching television or using computer/video games. Students heights and weights were objectively measured. We applied principal component analysis (PCA) to derive the components of self-esteem, and multilevel, multivariable logistic regression to quantify associations of diet quality, PA, sedentary behaviour and body weight with these components of self-esteem.

**Results:**

PCA identified four components for self-esteem: self-perception, externalizing problems, internalizing problems, social-perception. Influences of health behaviours and body weight on self-esteem varied across the components. Better diet quality was associated with higher self-perception and fewer externalizing problems. Less PA and more use of computer/video games were related to lower self-perception and social-perception. Excessive TV watching was associated with more internalizing problems. Students classified as obese were more likely to report low self- and social-perception, and to experience fewer externalizing problems relative to students classified as normal weight.

**Conclusion:**

This study demonstrates independent influences of diet quality, physical activity, sedentary behaviour and body weight on four aspects of self-esteem among children. These findings suggest that school programs and health promotion strategies that target health behaviours may benefit self-esteem in childhood, and mental health and quality of life later in life.

## Background

Self-esteem is widely viewed as a person’s overall evaluation about self-worth or personal value (Rosenberg et al. 1995; Rosenberg 1965), and is considered an essential indicator for positive mental health and psychological well-being (Mann et al. 2004; Ryff 1989). Health-related quality of life (HRQOL) is defined as a multidimensional construct that includes physical, social and emotional functioning, as well as psychological well-being (Spieth and Harris 1996). Self-esteem questions have been frequently used as one of the key components in a multidimensional HRQOL measure to assess self-esteem, mental health and psychosocial well-being among children and youth in both patient and general population groups (Ravens-Sieberer and Bullinger 1998; Varni et al. 2006). Low self-esteem has been linked to serious mental health consequences, such as depression, anxiety, suicidal ideation, violent behaviour and substance use in children and adolescents (Mann et al. 2004; Orth et al. 2014; McGee et al. 2001; McClure et al. 2010). Low self-esteem in children may also influence their cognitive development and school academic performance (Davies and Brember 1999; Baumeister et al. 2003). This is because children with higher self-esteem may set for themselves higher learning goals for success, and be more confident to deal with hard questions in their studies (Davies and Brember 1999). Higher self-esteem may also help them to overcome negative feelings (e.g., anxiety, incompetence) when they encounter failure in achievement tests (Baumeister et al. 2003).

Health behaviours such as poor diet quality, lack of physical activity (PA), and sedentary behaviour have been identified as risk factors for low self-esteem in children and youth (Wang and Veugelers 2008; Tin et al. 2012; Swing et al. 2010; Biddle and Asare 2011). Previous studies have demonstrated that increased PA has a beneficial effect on self-esteem in children and adolescents (Wang and Veugelers 2008; Biddle and Asare 2011). Sedentary behaviour, typically characterized as increased use of screen-based media such as watching television or playing computer/video games, are associated with lower self-esteem (Tin et al. 2012; Swing et al. 2010). However, these earlier studies have focused on associations between self-esteem and a single health behaviour (e.g., physical activity or television viewing). It remains unclear whether diet quality, physical activity and sedentary behaviours are independent predictors for self-esteem in children when these multiple exposures are considered simultaneously, and when the potential confounding effects of demographic covariates are controlled. Furthermore, very few studies have examined influences of diet quality on self-esteem using childhood population-based data (Wang and Veugelers 2008).

The assessment of self-esteem among children is challenging as there is little consensus on what attributes and constructs for self-esteem should be included and consideration should be given to the cognitive abilities of children (Rosenberg et al. 1995; Davis-Kean and Sandler 2001). Self-esteem can be conceptualized as both a global attribute (uni-dimensional construct) of self-worth and as a domain or component-specific (multi-dimensional construct) measure of self-evaluation (Rosenberg et al. 1995; Rosenberg 1965; Piers and Harris 1964; Danielsen et al. 2012; Israel and Ivanova 2002). Different instruments have been developed to assess self-esteem among children and youth (Rosenberg et al. 1995; Harter 1985; Marsh and O’Neill 1984). These commonly consist of multiple domains and a global attribute. For example, the Harter Self-Perception Profile contains five domains and a global self-esteem score that is computed by aggregating the domain scores (Harter 1985). The mostly widely used Rosenberg Self-Esteem Scale includes 10 items (Rosenberg 1965), with multiple factor structures of the scale having previously been examined (McKay et al. 2014; Donnellan et al. 2016). However, previous research has mostly evaluated self-esteem through a global composite measure, rather than considering its sub-components and research examining risk factors for self-esteem on specific domains among children and youth is limited (Danielsen et al. 2012; Israel and Ivanova 2002; Griffiths et al. 2010). A significant association between a risk factor and global self-esteem may not represent the influence of its underlying domain-level self-esteem, such as body-esteem or physical appearance, behavioral problems (e.g., being either a bully or a bully-victim) or internalizing problems (e.g., feeling worried or sad). Assessment of self-esteem at different sub-components allows us to elucidate a more nuanced picture of relations between health behaviours and self-esteem. Understanding how health behaviours influence specific aspects of self-esteem is important in developing population health intervention programs to enhance self-esteem and mental health in children and adolescents.

As the prevalence of childhood obesity continues to be high (Skinner et al. 2016; Rodd and Sharma 2016), prevention continues to be a public health priority and new initiatives are to be considered by public health decision makers. Adverse consequences of obesity for mental health, including the risk of lower self-esteem, internalizing and externalizing problems in children, have attracted increasing attention (Halfon et al. 2013; Franklin et al. 2006). While the relationship between childhood obesity and low self-esteem has been previously investigated (Franklin et al. 2006; Wardle and Cooke 2005), the pattern of associations between body weight status and specific self-esteem outcomes remains unclear as most previous studies focused on analyzing self-esteem as a global outcome (Danielsen et al. 2012). There is a need to assess relationships between childhood body weight and attributes of self-esteem. For example, previous studies revealed that overweight children are more likely to report lower physical appearance or physical self-esteem, with lower perceived physical self-esteem in girls compared with boys (Danielsen et al. 2012; Israel and Ivanova 2002). A specific interest is to examine which aspects of self-esteem are influenced by weight status when multiple components of this outcome are analysed simultaneously, and whether the associations between body weight and specific self-esteem are independent of health behaviours and demographic characteristics.

 The present study aimed to investigate the principal components of self-esteem and to identify the health behavioural determinants of these self-esteem components among grade five students aged 10–11 years.

## Methods

### The survey

The Children’s Lifestyle and School Performance Study (CLASS) (2003) is a population-based survey in the province of Nova Scotia (NS) in Canada that evaluates the relationships between nutrition, physical activity, sedentary behaviour and mental health and school performance of children (http://www.nsclass.ca). Out of 291 elementary schools in NS, 282 (96.9 %) participated in the study. Of the 5517 grade five students within participating schools who received parental consent to participate, 5200 students completed the surveys. The average participation rate per school was 51.1 % (Veugelers and Fitzgerald 2005a).

CLASS consisted of a home survey that was completed by the parents; a Canadian version of the Harvard Youth/Adolescent (food frequency) Questionnaire (YAQ) (Rockett et al. 1995), administered to the students in the schools by study assistants, and a measurement of student height and weight. The home survey collected information on student place of residency, gender, household income, parental education level, and questions on students’ PA and screen use time on watching television or playing computer/video games. Standing height was measured by a study assistant to the nearest 0.1 cm; body weight was measured to the nearest 0.1 kg on calibrated digital scales.

### Exposures

The main exposures of interest in this analysis are diet quality, physical activity, sedentary behaviour (or screen time) and body weight. On the basis of students’ nutrient intake and dietary information from the YAQ and Canadian Nutrient Files (Health Canada 2007), we calculated intake of nutrients and daily energy intake, as well as number of daily servings of fruits and vegetables. We then calculated a Diet Quality Index (DQI) score based on the composite measure, DQI-international (DQI-I) (Kim et al. 2003). The DQI-I constitutes four components: variety, adequacy, moderation and overall balance of the diet (Kim et al. 2003). The DQI-I score ranges between 0 and 100, with a higher score indicating a better diet quality. We divided the DQI-I score into tertiles (low, middle, high) in the analysis.

Parents were asked questions about the frequency that their children engaged in playing sports or doing PAs with or without a coach, with PA level grouped into four categories: *never or almost never, about once a month, about once a week, more than once a week*. Parents also reported on the frequency that their children engaged in sedentary behaviours out of school hours. Screen-based sedentary time was defined as daily number of hours spent on watching television, playing computer or video games: <*1, 1*–*2, 3*–*4,* ≥*5* *h/day*.

We used the age- and gender-specific body mass index (BMI) cut-off points for children established by the International Obesity Task Force (Cole et al. 2000), categorized as *normal weight, overweight and obesity*.

### Self-esteem outcomes

A variety of instruments have been used to assess self-esteem among different age groups of children and youth (Statistics Canada 1995; Harter 1985; Marsh and O’Neill 1984; Rosenberg 1965). Based on these instruments, we derived 11 items suitable for the grade five students in the present study who are primarily 10 and 11 years of age. The questions cover students’ self-perception about physical appearance/body-esteem, satisfaction with self, outlook for the future, emotional attributes, peer relationships, aggressive behaviour or bully-victim status, and attention problems. The items for self-esteem included: (1) I feel like I do not have any friends; (2) My future looks good to me; (3) I like the way I look; (4) I like myself; (5) I feel unhappy or sad; (6) I worry a lot; (7) I cry a lot; (8) I get into physical fights; (9) I am bullied by other kids; (10) I bully other kids. (11) I have trouble paying attention. Response options for each of the items were: *Never or almost never, Sometimes and Often or almost always*. We scored these responses such that a higher score represented higher self-esteem. A global self-esteem score was computed by summing the 11 item scores. Our previous work has demonstrated validity and reliability of the self-esteem scale as a global measure (Wang and Veugelers 2008). The reliability coefficient (Cronbach’s alpha) of the 11 items of self-esteem scale was 0.69 in the sample (n = 4945) with complete self-esteem information.

### Socio-demographic covariates

Students’ gender, household income, highest parental educational level, and residence (urban vs. rural) were included as potential confounders in the regression analyses. Household income was collapsed into 4 levels: *$0–$20,000, $20,001*–*$40,000, $40,001*–*$60,000,* >*$60,000*. Parental education attainment was categorised as *Secondary school or lower, College, University or above*, and residency was classified as *Urban or Rural,* based on postal code.

### Statistical analysis

Descriptive analyses were conducted to describe the distribution of socio-demographic variables, diet quality, body weight, physical activity and sedentary behaviour using weighted proportions. Mean and standard deviation (SD) were reported for continuous variables and percentages for categorical variables. Principal component analysis (PCA) with varimax (orthogonal) rotation was applied to the 11 self-esteem questions to extract the principal components that maximize the proportion of variance explained in the total variance. The number of components was determined by examination of the percentage of variance explained by the components, the eigenvalue, factor loadings and scree plot of the components. The items with factor loadings ≥0.3 were retained for the corresponding components. The predicted scores for each of the chosen components were computed for the individual students. Subsequently, we dichotomized the estimated component score and used it as component-level self-esteem outcomes in the regression analysis. The binary variable for self-esteem was generated by the quantiles with an equal size of two groups.

The Kaiser–Meyer–Olkin (KMO) measure of sampling adequacy (Kaiser 1970) and Bartlett’s test of sphericity (Bartlett 1950) were used to assess suitability of the principal component analysis. A KMO value larger than 0.5 and statistical significance (p < 0.05) for the Bartlett’s test indicate that use of PCA is appropriate.

Multilevel multivariable logistic regressions were used to investigate the association between diet quality, PA, sedentary behaviour, body weight and self-esteem outcomes, adjusting for the effect of confounders: gender, household income, parental educational attainment, place of residency and other self-esteem components. Both global and component-level self-esteem outcomes were evaluated in the logistic regression models. As the summed score of the self-esteem items (ranges between 11 and 33) was not normally distributed, we dichotomized the global self-esteem score. Low global self-esteem was defined as a score less (global score <28) than the overall average score. Model parameters were expressed as odds ratios (ORs) for low self-esteem between comparison groups of the independent variables. Multilevel regression models accommodate the nested structure of the school data in which student observations are nested within their schools.

Of the 4945 students who had complete information on self-esteem, 27 students had missing diet quality information, leaving 4918 observations available in the analysis for the association between the lifestyle factors and self-esteem. Missing values for other covariate variables were considered as separate covariate categories in the regression analysis but their estimates are not presented.

As the 2003 CLASS participation rate in residential areas with lower estimates of household income was slightly lower than the average, response weights were calculated to overcome potential non-response bias and to obtain provincial population estimates for grade five students in Nova Scotia (Veugelers and Fitzgerald 2005b). The Stata/IC 14 statistical software package (Stata Corp., College Station, TX, USA) was used for the data analysis (StataCorp 2015).

The 2003 Children’s Lifestyle and School Performance Study including the parental consent procedures and the analysis of the present study were approved by the Health Sciences Human Research Ethics Board of Dalhousie University and the Health Research Ethics Board at the University of Alberta.

## Results

### Principal components

**Fig. 1:**
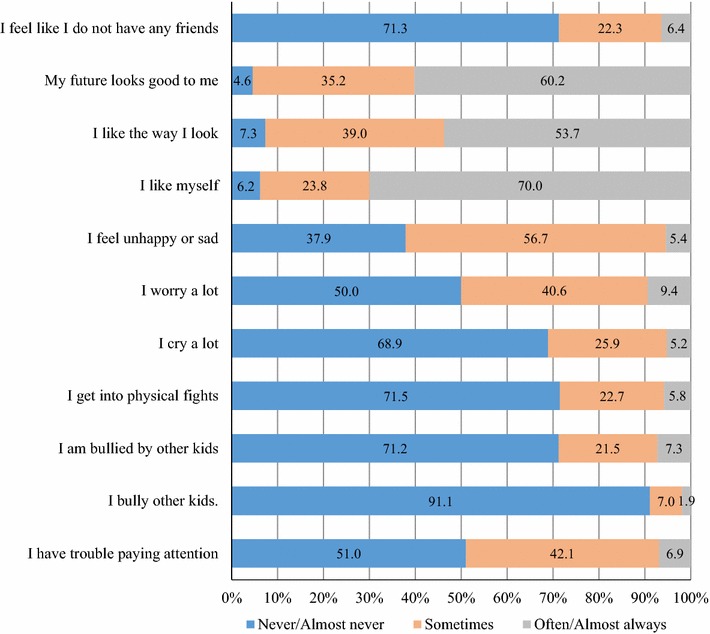
Frequency distribution (%) of responses to the self-esteem items (n = 4918)

Frequency distribution for the responses to the 11 self-esteem questions is presented in Fig. 1. The KMO value is 0.76. The Bartlett’s test of sphericity was significant (χ^2^ = 6873.25, *df* = 55, p < 0.01), suggesting that the application of PCA is suitable. Principal component analysis with subsequent varimax rotation reduced the questions on self-esteem to four components explaining 57.3 % of the total variance, with an eigenvalue greater than one for the first three components (Table 1). We labelled the four components of self-esteem as self-perception, externalizing problems, internalizing problems and social-perception (Table 1). The first component, self-perception, explained 24.9 % of the total variance before rotation. The 4th component, social-perception, has an eigenvalue of 0.9, explaining 7.9 % in total variance. Factor loadings ranged between 0.33 and 0.75 for the respective components. The first three components explained only 49.4 % of the total variance. The results suggest that a four component solution was better than retaining of three components.

**Table 1:** Principal component analysis for the 11-item self-esteem scale (n = 4945), the 2003 Children’s Lifestyle and School Performance Study, Nova Scotia

Component	Item description	Factor loading	Eigenvalue	Variance explained (%)
1. Self-perception	2. My future looks good to me	0.52	2.7	24.9
	3. I like the way I look	0.59		
	4. I like myself	0.61		
2. Externalizing problems	8. I get into physical fights	0.63	1.4	12.9
	10. I bully other kids.	0.66		
	11. I have trouble paying attention	0.33		
3. Internalizing problems	5. I feel unhappy or sad	0.44	1.3	11.6
	6. I worry a lot	0.61		
	7. I cry a lot	0.65		
4 Social-perception	1. I feel like I do not have any friends	0.75	0.9	7.9
	9. I am bullied by other kids	0.50		

Of the four components, self-perception is closely related to an attribute of body-esteem and hopes for the future; the component of externalizing problems represents an aspect of self-esteem as it relates to the expression of aggressive behaviours, including bullying other children, engagement in physical fights and having trouble paying attention; the internalizing problems component is closely related to a domain of unhappiness and anxiety; and the social-perception component represents a facet of social relationships that links to being a bully-victim and friendships with peers (Table 1).

### Associations between health behaviours, body weight and self-esteem

Table 2 presents the distribution of the sample by the demographic and health behaviour variables, and the multivariable logistic regression results for self-esteem. The prevalence of overweight and obesity was 23.3 and 9.9 % respectively. The mean score for global self-esteem was 28.31 (SD = 3.21). Higher diet quality, and an increased level of PAs (e.g., more than once a week) were associated with higher global self-esteem. Excessive TV viewing and excessive weight (obese) status was correlated with lower global self-esteem. The logistic regression results for determinants of the self-esteem components were depicted for each component respectively.

**Table 2:** Odds ratio for low self-esteem by the health behaviours, body weight and socio-demographic variables among grade five students (n = 4918), the 2003 Children’s Lifestyle and School Performance Study, Nova Scotia

Variable	% students	Self-perception	Externalizing problems	Internalizing problems	Social-perception	Global self-esteem
		OR (95 % CI)	OR (95 % CI)	OR (95 % CI)	OR (95 % CI)	OR (95 % CI)
Diet quality index						
Low tertile	–	1.0	1.0	1.0	1.0	1.0
Middle tertile	–	0.89 (0.76, 1.03)	*0.83 (0.71, 0.98)*	0.95 (0.81, 1.12)	0.96 (0.83, 1.11)	*0.81 (0.69, 0.94)*
High tertile	–	*0.82 (0.70, 0.95)*	*0.81 (0.69, 0.94)*	1.03 (0.89, 1.20)	0.93 (0.80, 1.08)	*0.80 (0.70, 0.92)*
Play sports/PAs without coach						
Never or almost never	4.7	1.0	1.0	1.0	1.0	1.0
About once a month	6.2	0.87 (0.58, 1.32)	1.25 (0.81, 1.94)	0.78 (0.52, 1.17)	0.82 (0.55, 1.22)	0.72 (0.48, 1.09)
About once a week	21.7	0.87 (0.61, 1.23)	1.23 (0.86, 1.77)	0.98 (0.66, 1.43)	0.80 (0.57, 1.12)	0.79 (0.57, 1.10)
More than once a week	67.4	0.79 (0.57, 1.10)	1.41 (1.00, 2.00)	0.82 (0.57, 1.17)	*0.70 (0.52, 0.96)*	*0.72 (0.54, 0.98)*
Play sports/PAs with coach						
Never or almost never	27.6	1.0	1.0	1.0	1.0	1.0
About once a month	6.5	1.18 (0.90, 1.56)	1.22 (0.90, 1.65)	0.86 (0.64, 1.16)	1.04 (0.78, 1.39)	1.22 (0.90, 1.67)
About once a week	30.6	1.06 (0.90, 1.25)	0.95 (0.78, 1.15)	0.96 (0.80, 1.14)	0.93 (0.79, 1.09)	0.99 (0.82, 1.19)
More than once a week	35.3	*0.83 (0.70, 0.98)*	0.98 (0.81, 1.19)	0.92 (0.77, 1.10)	0.85 (0.71, 1.01)	*0.75 (0.62, 0.90)*
Using computer/video games						
<1 h/day	53.9	1.0	1.0	1.0	1.0	1.0
1–2 h/day	39.9	*1.17 (1.02, 1.33)*	1.06 (0.91, 1.22)	0.93 (0.82, 1.07)	0.98 (0.85, 1.14)	0.99 (0.86, 1.13)
≥3 h/day	6.2	*1.55 (1.12, 2.15)*	0.91 (0.66, 1.25)	0.86 (0.65, 1.15)	1.00 (0.73, 1.35)	1.03 (0.78, 1.37)
Watching TV						
<1 h/day	13.9	1.0	1.0	1.0	1.0	1.0
1–2 h/day	56.8	0.88 (0.73, 1.07)	1.00 (0.81, 1.23)	1.20 (0.99, 1.46)	0.98 (0.79, 1.21)	0.99 (0.81, 1.22)
3–4 h/day	25.8	0.88 (0.71, 1.08)	1.16 (0.92, 1.47)	1.18 (0.95, 1.46)	1.03 (0.81, 1.30)	0.98 (0.78, 1.24)
≥5 h/day	3.5	1.01 (0.66, 1.53)	1.16 (0.78, 1.73)	*1.68 (1.11, 2.55)*	1.12 (0.75, 1.69)	*1.92 (1.31, 2.80)*
Body weight						
Normal weight	66.8	1.0	1.0	1.0	1.0	1.0
Overweight	23.3	*1.20 (1.03, 1.41)*	0.87 (0.73, 1.03)	1.13 (0.97, 1.32)	1.03 (0.88, 1.21)	1.15 (0.98, 1.36)
Obese	9.9	*1.55 (1.21, 1.98)*	*0.78 (0.61, 1.00)*	1.19 (0.97, 1.46)	*1.29 (1.02, 1.62)*	*1.42 (1.12, 1.79)*
Gender						
Boys	49.2	1.0	1.0	1.0	1.0	1.0
Girls	50.8	*1.29 (1.13, 1.49)*	*0.30 (0.26, 0.35)*	*1.44 (1.27, 1.65)*	0.93 (0.81, 1.05)	*0.79 (0.70, 0.90)*
Household income						
<$20,000	12.1	1.0	1.0	1.0	1.0	1.0
$20,001–$40,000	22.8	0.81 (0.62, 1.07)	1.00 (0.77, 1.30)	1.03 (0.80, 1.32)	0.86 (0.68, 1.09)	*0.75 (0.59, 0.94)*
$40,001–$60,000	26.1	0.91 (0.71, 1.16)	*0.72 (0.56, 0.92)*	0.95 (0.74, 1.23)	0.82 (0.64, 1.05)	*0.57 (0.45, 0.73)*
>$60,000	39.0	*0.75 (0.59, 0.95)*	*0.66 (0.51, 0.86)*	0.94 (0.72, 1.22)	*0.78 (0.62, 0.97)*	*0.52 (0.41, 0.65)*
Parent education						
Secondary or less	30.3	1.0	1.0	1.0	1.0	1.0
College	37.4	0.91 (0.79, 1.05)	1.00 (0.85, 1.18)	0.95 (0.82, 1.11)	1.01 (0.86, 1.18)	0.97 (0.82, 1.15)
University or above	32.3	*0.79 (0.67, 0.94)*	*0.78 (0.64, 0.95)*	0.92 (0.77, 1.11)	0.84 (0.71, 1.00)	*0.66 (0.53, 0.81)*
Residency						
Rural	32.8	1.0	1.0	1.0	1.0	1.0
Urban	67.2	0.90 (0.77, 1.05)	*1.21(1.03, 1.42)*	1.02 (0.89, 1.17)	*0.86 (0.75, 1.00)*	0.95 (0.81, 1.12)
Self-esteem component						
Self-perception	–	–	*1.74 (1.52, 2.00)*	*1.49 (1.32, 1.69)*	*1.95 (1.72, 2.22)*	–
Internalizing problems	–	*1.74 (1.52, 2.00)*	–	*1.15 (1.00, 1.32)*	*2.67 (2.33, 3.06)*	–
Externalizing problems	–	*1.49 (1.32, 1.68)*	*1.16 (1.01, 1.33)*	–	*2.60 (2.31, 2.94)*	–
Social-perception	–	*1.96 (1.73, 2.23)*	*2.70 (2.35, 3.10)*	*2.62 (2.32, 2.95)*	–	–

Italicised values for OR (odds ratio) with 95 % CI (confidence interval) indicate p < 0.05. Reference group for self-esteem is the high self-esteem category. Low self-esteem for the global self-esteem was defined as scores <28. The regression model was mutually adjusted for the variables in the table

#### Self-perception

Students with the highest diet quality had lower odds (OR 0.82, 95 % CI 0.70–0.95) of reporting low self-esteem as it relates to self-perception. Relative to never playing sports or PAs with a coach, children who play sports or PAs with a coach more than once a week were less likely to have a lower self-perception. Using computer or video games for more than 1 h per day was significantly associated with lower self-perception. Overweight (OR 1.20, 95 % CI 1.03–1.41) and obese (OR 1.55, 95 % CI 1.21–1.98) status were associated with lower self-esteem as it relates to self-perception. Females and students in the lowest family income and parental education level reported lower self-perception than males and students with higher household income and more parental education.

#### Externalizing problems

Students with higher diet quality were less likely to report negative behaviours such as getting into physical fights, bullying other kids or having trouble paying attention (high vs. low tertile: OR 0.81, 95 % CI 0.69–0.94). Boys had higher odds of experiencing externalizing problems than girls.

#### Internalizing problems

TV viewing is negatively associated with the attribute of internalizing problems indicated by feeling unhappy or sad, worried and crying. Students who watched TV for five or more hours per day had 68 % greater odds (adjusted OR 1.68, 95 % CI 1.11–2.55) of showing internalizing problems. Girls were 1.44 times more likely to feel unhappy or sad, to worry or cry a lot than boys (OR 1.44, 95 % CI 1.27–1.65).

#### Social-perception

Children playing sports or PAs without a coach more than once a week were less likely to rate problems on social-perception (OR 0.70, 95 % CI 0.52–0.96). Relative to students in lowest family income, and in rural residence, students with household income >$60,000 and living in an urban residency showed lower odds of being bullied or having a lack of friends.

## Discussion

To our knowledge, this is the first population-based study that has analysed specific self-esteem outcomes and their associations with multiple health behaviours, including diet quality, PA, sedentary behaviours and body weight in a large sample of Canadian school children. We found that diet quality, PA and sedentary behaviours are important determinants of self-esteem among children, and the effect of these factors varies across the four underlying components of self-esteem. These associations are independent of body weight status, socio-demographic background, and other mutual self-esteem aspects. After adjusting for other covariate effects, students who were classified as obese relative to healthy weight students had a higher likelihood of being bullied by other children or lack of friends, and of reporting lower self-perception.

Children and adolescents with low self-esteem are at greater risk of developing more severe mental health conditions or expressing suicidal ideation, and to engage in risky behaviours such as drug use or delinquency (Orth et al. 2014; Wild et al. 2004; Donnellan et al. 2005). This highlights the importance of studying how health-behaviours may impact self-esteem and to design effective population health intervention programs to protect or improve children’s self-esteem. In this study, we specifically examined the effect of diet quality, different types of PA and sedentary behaviours on the underlying components of self-esteem. The advantage of assessing specific self-esteem outcomes is that the relative importance of risk factors contributing to different facets of self-esteem can be quantified, and clustered patterns of individual self-esteem items can be identified. The current study confirms our previous findings that diet quality, PA and sedentary time are significant predictors for global self-esteem (Wang and Veugelers 2008), and further extends the observation that the magnitude of effects of the health behaviours differs across the self-esteem subcomponents. We observed that better diet quality has a beneficial effect on self-perception and is associated with fewer externalizing problems linked to negative behaviours like physical fights, bullying and attention problems. Children who reported to participate in unorganized sports (PA without a coach) also reported better self-esteem on the component of social-perception (which is measured by having friends or not, and by being bullied by peers). Children who reported to participate in organized sports (PA with a coach) reported better self-esteem on the component of self-perception (which is measured by the child’s self-perception and the perception of her/his future). The results suggest that school program and health promotion initiatives among children that improve healthy eating and active living, may also benefit self-esteem and help prevent aggressive behaviours such as bullying and attention problems. Canadian studies have shown that intervention programmes that implement multifaceted, comprehensive school health promotion framework integrating healthy eating and active living foster positive effects on students’ health behaviour and contribute to weight reduction (Veugelers and Fitzgerald 2005a; Fung et al. 2012). Few studies have explored effects of sedentary activities on self-esteem among children (Wang and Veugelers 2008; Tin et al. 2012). We found children who spent excessive time (5 or more hours daily) watching TV relative to those spending <1 h a day experienced lower self-esteem as it relates to internalizing problems, which could be a marker for anxiety and depression. The result is consistent with prior studies showing that more TV viewing is related to low self-esteem, anxiety and depression (Tin et al. 2012; Maras et al. 2015). A possible explanation for this association is that children who watch excessive hours of TV are less likely to experience social interactions with peers or family, which in turn could result in greater feelings of worry or anxiety (Tin et al. 2012). It may also be possible that some programs shown on TV (e.g., violence) may heighten a child’s experience of worry or sadness. Future research is needed to investigate the influence of these factors on self-esteem outcomes.

As previous research demonstrated associations between use of video games and TV and attention problems in children (Swing et al. 2010; Ozmert et al. 2002; Landhuis et al. 2007), we performed additional analysis to assess the association between TV viewing, use of computer or video games and the self-esteem question: *I have trouble paying attention.* The results show that children who spend more time in front of the TV reported more attention problems, which confirms the previous finding (Swing et al. 2010; Ozmert et al. 2002; Landhuis et al. 2007). We did not observed a significant relationship between use of computer or video games and attention problems after adjustment for the other exposure and demographic variables.

When comparing the results between the regression model for global self-esteem with those for components of self-esteem, distinct associations appeared. For example, while the regression model did not find a significant relationship between use of computer/playing video games and global self-esteem, a graded association between use of computer/playing video games and the self-esteem component self-perception was observed, whereby children with more time (≥1 h per day) spent on computer use or playing video games had poorer self-perception. This is similar to previous literature reporting that more electronic screen use is related to an elevated risk of low self-esteem and more mental well-being problems among children and adolescents (Yang et al. 2013; Racine et al. 2011). The regression model for global self-esteem failed to capture a significant effect of residency for self-esteem. The regression model for component-specific self-esteem indicated a significant difference in the externalizing problems between children of rural and urban residency. Although boys were more likely to experience lower overall self-esteem than girls, this gender disparity does not apply for all the components. Girls were more likely to report lower self-perception and more internalizing problems (Table 2).

A differential effect of childhood obesity for self-esteem was observed for two components, externalizing problems and social-perception pertaining to physical fights, bullying behaviour and being the victim of bullying respectively. Students who were classified as obese relative to healthy weight students were more likely to be bullied by other children rather than bullying other peers. This association has also been reported in other studies, thus placing children who are obese at higher risk for low self-esteem (Danielsen et al. 2012; Janssen et al. 2004; van Geel et al. 2014).

The contributions of socio-demographic factors to self-esteem observed in this study are seemingly consistent with those of earlier studies. For example, girls were less satisfied with their appearance (Danielsen et al. 2012; Israel and Ivanova 2002; Wild et al. 2004), more likely to feel sad and worried or to cry more than boys (Wu et al. 2012), while boys were more likely to be involved in aggressive behaviours such as getting into physical fights, bullying other children and having attention problems (Veenstra et al. 2005). Higher levels of family income and parental education are strong protective factors for self-esteem (Danielsen et al. 2012). The results underscore the importance of targeting school health promotion efforts towards children from socioeconomically disadvantaged communities, in order to promote and improve self-esteem.

As disagreements remain about the definition and theory of self-esteem, how self-esteem is measured and analysed among children and youth requires further research. An important issue in the assessment of self-esteem is whether it is conceptualized as a global unidimensional or as a multidimensional construct with subcomponents. Some researchers examined the outcomes of specific domains of self-esteem (Danielsen et al. 2012; Israel and Ivanova 2002; Wild et al. 2004; Marsh and O’Neill 1984), such as physical appearance, social perception (e.g., belief of how other people perceive them) and academic performance. This study contributes to the existing research on self-esteem by examination the relationship between the health behaviour and each of four independent components of self-esteem in a population-based sample of children. Future studies are needed to examine the relative importance of each self-esteem component for the overall evaluation of self-esteem, and to replicate the present finding in other study samples.

As self-esteem is extensively used as a health outcome in health-related quality of life or as a patient-reported outcome, health intervention programs for self-esteem improvement in children would be expected to benefit health-related quality of life as well. One study reported children who were being victims of bullying had lower HRQOL than non-victims (Beckman et al. 2016). Examination of the association between self-esteem and quality of life is beyond the objective of the present study. Future research warrant to address how each of the self-esteem component influence HRQOL among children and youth.

The large population-based sample that allowed for the adjustment of various confounders should be considered a strength of the present study. Strengths extend to the fact that we used measured heights and weights. We applied the widely used body mass index cut-offs for the classification of overweight and obesity by the International Obesity Task Force (Cole et al. 2000). Studies have shown that this method provides similar classification for overweight and a lower estimate for obesity relative to the WHO cut-points (Shields and Tremblay 2010; Wang and Wang 2002). Further, our analyses accommodated the sampling design and the hierarchical data structure, provided robust estimates representative for the population of grade five students of 10–11 years old for the province of Nova Scotia. However, as the CLASS data were collected in one period of time (2003), the analysis for this study do not allow for causal inference or temporality between the exposures of interest and self-esteem outcomes. Longitudinal studies are needed to confirm the present findings by assessing development or changes of self-esteem problems from childhood to early adulthood. Assessment of child physical activity and sedentary behaviours used parental self-report, which are prone to bias. However, we have previously reported that parents were able to provide accurate assessment for their child physical and sedentary activities (Sithole and Veugelers 2008). Objective measurement of physical activity, such as use of accelerometers, would provide more accurate physical activity data, though such objective method would be financially challenging in large population-based epidemiological studies. The assessment of dietary intake was also based on self-report, although the Harvard Food Frequency Questionnaire for Youth and Adolescents has been previously validated and is a widely used food frequency measure (Rockett et al. 1997). As we used data collected in 2003, our findings may not accurately apply to the present as children’s lifestyles continue to change. For example, children now spend more time being sedentary than children did in 2003 (Chinapaw et al. 2011).

## Conclusion

This study demonstrates independent influences of diet quality, physical activity and sedentary behaviour on four aspects of self-esteem among children. The results suggest that school programs and health promotion strategies may improve self-esteem in childhood and mental health later in life. Longitudinal studies are needed to confirm temporal relationships among health behaviours and self-esteem and possible subsequent mental health conditions including depression and attention deficit and hyperactivity disorder.
